# Analysis of Circadian Blood Pressure Patterns and Their Clinical, Metabolic, and Structural Correlates in Newly Diagnosed Hypertensive Patients

**DOI:** 10.3390/biomedicines14071491

**Published:** 2026-06-30

**Authors:** Kaya Özen, Süleyman Akkaya

**Affiliations:** 1 Department of Cardiology, Gazi Yasargil Training and Research Hospital, University of Health Sciences, 21070 Diyarbakır, Turkey; 2 Faculty of Law, Dicle University, 21280 Diyarbakır, Turkey; slymnkky1982@gmail.com

**Keywords:** hypertension, circadian blood pressure, left ventricular hypertrophy, left atrial diameter, triglyceride–glucose index, atherogenic index of plasma

## Abstract

**Background**: This study aimed to investigate the relationships of AHA/ACC blood pressure stages and circadian blood pressure patterns with clinical, metabolic, inflammatory, and structural echocardiographic parameters in newly diagnosed hypertensive (HT) patients, and to identify independent predictors of high-risk circadian disruptions. **Methods**: A total of 539 patients undergoing 24 h Ambulatory Blood Pressure Monitoring (ABPM) between 2022 and 2024 were retrospectively analyzed. Patients were grouped by nocturnal blood pressure dipping percentages and HT stages; laboratory markers (TyG index (Triglyceride–Glucose İndex), AIP (Atherogenic İndex of Plasma), SII (Systemic Immune-Inflammation Index), AISI (Aggregate Index of Systemic Inflammation), NLR (Neutrophil-to-Lymphocyte Ratio)) and echocardiographic data were evaluated via multivariable logistic regression. **Results**: The mean cohort age was 46.49 ± 13.98 years. Progressing HT stages were associated with significant increases in metabolic risk indicators: TyG index (*p* = 0.002) and AIP (*p* = 0.004). The prevalence of left ventricular hypertrophy (LVH) increased significantly from 7.3% in the normal group to 34.5% in Stage 2 HT (*p* < 0.001). The highest-risk “reverse-dipper” group had a significantly higher mean age (50.32 ± 14.55 years) than other groups (*p* < 0.001). In multivariable logistic regression, left atrial diameter (LAd) was the only common independent structural predictor of circadian disruption across all models (Extreme-Dipper OR: 1.19, *p* = 0.003; Non-Dipper OR: 1.11, *p* = 0.001). In the Reverse-Dipper model score analysis, both LAd (*p* < 0.001) and LVH (*p* = 0.001) demonstrated strong independent predictive potential. Inflammatory indices (SII, AISI, NLR) showed no independent predictive value (*p* > 0.05). **Conclusions**: Advanced HT stages and disrupted circadian rhythms are strongly associated with metabolic impairment, left atrial dilatation, and LVH. In older hypertensive patients presenting with left atrial enlargement and LVH, echocardiographic screening and close ABPM are crucial for early “reverse-dipper” pattern detection.

## 1. Introduction

Hypertension (HT) is a major public health problem worldwide, accounting for approximately 10.4 million deaths annually, and is recognized as the most fundamental risk factor for cardiovascular diseases [1]. Uncontrolled HT is directly associated with increased morbidity and mortality rates. Thus, HT represents a heterogeneous clinical entity in which circadian blood pressure patterns significantly influence cardiovascular prognosis. Although conventional measurements remain valuable in clinical practice, Ambulatory Blood Pressure Monitoring (ABPM) is a much more effective tool than manual measurements for determining cardiovascular risk by detecting circadian variations [2]. In a normal circadian rhythm, a 10–20% decline in nocturnal blood pressure values compared to daytime values is defined as a “dipper” HT pattern. Conversely, a reduction of less than 10% is termed “non-dipper”, while an increase in nocturnal blood pressure above daytime values is classified as “reverse dipper”, which represents the highest-risk profile [3].

Disruptions in the circadian rhythm, particularly the ‘reverse dipper’ pattern, have been reported to strongly correlate with advanced target organ damage and elevated cardiovascular risk [4]. Despite the clinical utility of ABPM, constraints regarding its cost and accessibility have heightened the need for cost-effective and readily accessible biomarkers capable of predicting high-risk circadian disruptions. Circadian BP dysregulation is directly associated with cardiac structural remodeling, an indicator of target organ damage. The literature demonstrates that blunted or reversed nocturnal BP dipping results in an increased left ventricular mass index, changes in relative wall thickness, and left ventricular hypertrophy [5,6]. Furthermore, impairments in left atrial diameter and left atrial mechanics have been correlated with the loss of circadian rhythm, being particularly more pronounced in reverse-dipper patients [7,8].

Beyond traditional cardiovascular risk factors, the role of novel biomarkers reflecting insulin resistance and chronic inflammation in the pathogenesis of HT has been intensively investigated in recent years. The triglyceride–glucose index and the atherogenic index of plasma, as robust indicators of atherogenicity and metabolic dysfunction, hold high potential for predicting circadian rhythm disturbances [9,10]. Despite these data in the current literature, the integrated impact of these metabolic and inflammatory indices, along with cardiac geometric parameters, in identifying the reverse-dipper pattern in newly diagnosed hypertensive individuals has not yet been fully elucidated. This study aims to contribute to the early detection of high-risk patient profiles by comprehensively examining the effects of age, cardiac morphology, and metabolic/inflammatory markers on circadian rhythm disturbances.

## 2. Materials and Methods

### 2.1. Study Population

This study is a retrospective analysis of 539 patients who presented to the cardiology clinic with a preliminary diagnosis of hypertension and subsequently underwent 24 h ambulatory blood pressure monitoring between 2022 and 2024. Demographic, laboratory, and echocardiographic data of the patients were obtained from the hospital electronic medical record system. The exclusion criteria were defined as follows: chronic kidney disease (estimated glomerular filtration rate [eGFR] < 60 mL/min/1.73 m^2^), signs of active infection (erythrocyte sedimentation rate > 20 mm/h, C-reactive protein [CRP] > 3 mg/L, white blood cell count > 11 × 10^9^/L), known coronary artery disease, heart failure, thyroid dysfunction, anemia, sleep apnea syndrome, and prior use of antihypertensive medication. The study was conducted in accordance with the Declaration of Helsinki and was approved by the Clinical Research Ethics Committee of Health Sciences University, Di-yarbakır Gazi Yaşargil Training and Research Hospital, Turkey (approval number: 6, date of approval: 9 January 2026).

### 2.2. Ambulatory Blood Pressure Monitoring

Twenty-four-hour ABPM was performed using a portable digital recording device (Schiller MT-300 BP (Schiller AG, Baar, Switzerland)). Measurements were obtained at 15 min intervals during the daytime (06:01–22:00) and at 30 min intervals during the nighttime (22:01–06:00). Hypertension was defined as a 24 h average systolic blood pressure (SBP) > 120 mmHg and/or diastolic blood pressure (DBP) > 80 mmHg. Patients were categorized using two distinct approaches. First, based on the AHA/ACC hypertension guidelines, individuals were classified into four groups: Normal (BP < 120/80 mmHg), Elevated (BP 120–129/<80 mmHg), Stage 1 Hypertension (BP 130–139/80–89 mmHg), and Stage 2 Hypertension (BP ≥ 140/90 mmHg). The 191 individuals in the “Normal” category served as a normotensive control group to evaluate relative differences in inflammatory indices and echocardiographic parameters across the hypertension stages. Second, patients were categorized according to the percentage of nocturnal blood pressure decline into four circadian patterns: dipper (10–20% decline), non-dipper (<10% decline), reverse dipper (no de-cline or an increase), and extreme dipper (>20% decline).

### 2.3. Laboratory and Index Analyses

Blood samples were obtained from all patients after a 12 h fasting period. In order to assess the participants’ metabolic and inflammatory status, the following composite indices which have been previously defined in the literature and proven to be valid were used. These indices are not original parameters proposed by us, but were calculated in accordance with the formulas specified by their original authors:

The Systemic Immune-Inflammation Index (SII) was defined as [11]:

SII=Neutrophils×Platelets/Lymphocytes

The Aggregate Index of Systemic Inflammation (AISI) was calculated as [12]:

AISI=Neutrophils×Monocytes×Platelets/Lymphocytes

The Neutrophil-to-Lymphocyte Ratio (NLR) was calculated as [13]:

NLR=Neutrophils/Lymphocytes

The Triglyceride–Glucose (TyG) index was calculated using the following formula [14]:

TyG Index=ln[Fasting Triglycerides (mg/dL)×Fasting Glucose/2]

Additionally, the Atherogenic Index of Plasma (AIP) was calculated as the logarithmically transformed ratio of triglycerides to high-density lipoprotein cholesterol (TG/HDL-C) [15]:

AIP=log_{10}{Triglycerides/HDL−C}

### 2.4. Echocardiography

Echocardiographic examinations were performed using a 2.5 MHz transducer ((Philips Healthcare, Boston, MA, USA). The left ventricular ejection fraction (LVEF) was calculated via the modified Simpson’s method. İnterventricular septal thickness in diastole (IVSD), and left ventricular posterior wall diameter in diastole (LVPWd)), and left atrial diameter (LAd) were measured in the parasternal long-axis view. Additionally, relative wall thickness (RWT) was calculated as an indicator of left ventricular geometric remodeling using the following formula: RWT = (2 × LVPWd)/LVEDD (left ventricular end-diastolic diameter) [16].

### 2.5. Statistical Analysis

Statistical analyses were performed using IBM SPSS Statistics software (Version 26.0; IBM Corp., Armonk, NY, USA). Continuous variables were compared using One-Way Analysis of Variance (ANOVA) followed by Duncan’s post hoc multiple comparison test, while categorical variables were analyzed using the Chi-square (χ^2^) test. To identify the predictors of the reverse dipper pattern, univariable logistic regression analysis was initially performed, followed by a multivariable model incorporating the variables that demonstrated statistical significance. The results were presented as Odds Ratios (OR) with 95% Confidence Intervals (CI). A *p*-value of <0.05 was considered statistically significant.

## 3. Results

### 3.1. Clinical and Demographic Characteristics

The study cohort was established through a retrospective evaluation of 539 patients who presented to the cardiology outpatient clinic with a preliminary diagnosis of hypertension and underwent 24 h ambulatory blood pressure monitoring. The mean age of the entire study population was found to be 46.49 ± 13.98 years, whilst the median age of the participants was 46.00 years (range: 17.00–82.00 years). When the gender distribution was examined, 61.2% of the cohort consisted of female patients (*n* = 330) and 38.8% of male patients (*n* = 209). According to clinical history data, 13.7 per cent of patients (*n* = 74) had diabetes mellitus and 13.0 per cent (*n* = 70) had a history of previous cardiovascular disease (Table 1).

In the laboratory analyses, the patients’ baseline biochemical and haematological profiles were examined. The mean total cholesterol level was measured as 193.90 ± 38.26 mg/dL, the LDL-C level as 115.60 ± 31.57 mg/dL and the fasting glucose level as 103.56 ± 37.62 mg/dL. Among the inflammatory markers, the mean CRP level was 4.10 ± 4.60 mg/L, whilst the white blood cell count was 8.05 ± 2.19 × 10^3^/μL (Table 2).

In a comparative analysis based on the AHA/ACC stages of hypertension, the age and gender distributions showed statistically significant differences between the groups (*p* = 0.010 and *p* < 0.001). In particular, it was found that the prevalence of left ventricular hypertrophy, which was 7.3% in the normal group, increased significantly to 34.5% in the Stage 2 HT group (*p* < 0.001). In contrast, no statistically significant difference was found between the stages in terms of the presence of diabetes mellitus (DM) and previous cardiovascular history (*p* > 0.05) (Table 3).

The hourly ABPM recordings of 539 patients were re-analysed to calculate the daily mean, maximum and minimum values for systolic blood pressure, diastolic blood pressure and heart rate. Accordingly, the daily mean systolic blood pressure (SBP) was found to be 126.61 ± 15.83 mmHg, the mean diastolic blood pressure (DBP) 75.14 ± 10.24 mmHg, and the mean heart rate 78.29 ± 9.48 bpm. These daily averages, which correspond to MESOR (adjusted to the 24 h average) values, were compared with the maximum values (SBP: 130.23 ± 16.39 mmHg, DBP: 77.77 ± 10.80 mmHg, heart rate: 81.45 ± 10.05 bpm), the magnitude of the 24 h blood pressure burden and the associated cardiovascular stress become apparent. The minimum values representing the night-time period (SBP: 114.57 ± 28.68 mmHg, DBP: 66.80 ± 17.16 mmHg, heart rate: 67.93 ± 16.86 bpm) were combined with the circadian data and formed part of the key findings of this study. When our data were analysed in terms of circadian patterns, significant differences were observed between groups in the age distribution according to circadian blood pressure patterns (*p* < 0.001). The mean age of patients with a reverse-dipper pattern (50.32 ± 14.55 years) was found to be significantly higher than that of the dipper (42.15 ± 13.15 years) and extreme-dipper (38.42 ± 13.01 years). This finding highlights the strong association between advanced age and the reverse-dipper circadian rhythm (Table 4).

### 3.2. Echocardiographic Findings in the Groups

When the echocardiographic parameters of the study population were analyzed, structural variations were detected across both blood pressure stages and circadian patterns. According to the AHA/ACC stages, the mean left atrial diameter (LAd) was 34.27 mm in the Normal group, whereas this value reached its highest level in the High blood pressure stage at 36.06 mm. Interventricular septal thickness in diastole (IVSD) was measured as 10.71 mm in the High blood pressure stage and 10.59 mm in the Stage 1 HT group.

In the evaluation based on circadian patterns, the “Non-Dipper” group was observed to have the largest left atrial diameter (36.48 mm) and the highest relative wall thickness (0.41). While the IVSD was 10.49 mm in the “Reverse-Dipper” group, this value was recorded as 10.11 mm in the “Dipper” group. In patients with an “Extreme-Dipper” pattern, the mean left atrial diameter and RWT value were determined as 33.21 mm and 0.38, respectively. These findings demonstrate that circadian rhythm disruption (particularly Non-Dipper and Reverse-Dipper patterns) is closely associated with left ventricular geometric remodeling and left atrial dilatation (Table 5).

### 3.3. Metabolic, Inflammatory, and Structural Variables Associated with Hypertension Stages and Circadian Blood Pressure Patterns

In a comparative analysis based on the AHA/ACC stages of hypertension, statistically significant differences were observed in terms of metabolic risk indices and blood pressure regulation parameters.

Increase in Metabolic Indices: The Triglyceride–Glucose Index, an indirect marker of insulin resistance, showed a significant upward trend, rising from 8.79 ± 0.70 in the Normal group to 9.22 ± 0.78 in the Stage 2 HT group. Similarly, the Atherogenic Plasma Index, an important marker of cardiovascular risk, increased gradually as the stage of hypertension progressed, reaching its highest value in the Stage 2 HT group (0.52 ± 0.33).

Impaired Blood Pressure Regulation: The percentage decrease in systolic blood pressure from day to night (Decrease in Systolic BP %) exhibited a progressive decline across the AHA/ACC stages, falling from 10.37 ± 6.90 in the Normal group to 8.72 ± 7.91, then 8.35 ± 7.73, and ultimately 7.71 ± 7.15 in the Stage 2 HT group. This finding quantitatively demonstrates that as the blood pressure stage advances, patients become increasingly deprived of the protective hypotensive effect of sleep, and their autonomic blood pressure regulation progressively deteriorates (Table 6).

In the multivariate logistic regression analysis conducted to identify independent risk factors predicting different circadian blood pressure patterns, LAd was identified as the strongest structural parameter and remained statistically significant across all models. In predicting the Extreme-Dipper pattern, LAd (OR: 1.19, *p* = 0.003), AIP (OR: 0.04, *p* = 0.016) and the percentage decrease in systolic blood pressure (OR: 0.98, *p* = 0.001) were identified as independent predictive factors. However, in the Non-Dipper model, only LAd (OR: 1.11; *p* = 0.001) demonstrated a statistically significant predictive value. When switching to the Reverse-Dipper model, score analyses of out-of-model variables showed that LAd (*p* < 0.001), the percentage reduction in systolic blood pressure (*p* < 0.001) and the presence of LVH (*p* = 0.001) possessed very strong predictive potential. In particular, whilst inflammation indices such as AISI, NLR and SII showed differences in some groups in univariate analyses, none of them were identified as independent predictors in any of the multivariate models (*p* > 0.05) (Table 7).

## 4. Discussion

Hypertension is the primary preventable cause of cardiovascular diseases and all-cause mortality [17]. While early diagnosis and treatment are essential to reduce morbidity, manual blood pressure measurements often remain insufficient in capturing circadian variation [18]. In this study, our main findings revealed that an increasing stage of hypertension is associated not only with a haemodynamic burden but also with marked metabolic impairment, characterised by the TyG index and AIP, as well as structural cardiac changes such as left atrial enlargement. In our study, it was found that the TyG index and AIP—which are indirect markers of insulin resistance—increased significantly as the AHA/ACC stages progressed (*p* = 0.002 and *p* = 0.004). It has been reported in the literature that TyG and AIP levels are significantly higher in patients with white-coat hypertension who exhibit a ‘non-dipper’ pattern, and that these metabolic biomarkers may help to predict circadian rhythm disorders [10]. Similarly, other studies have argued that the AIP is one of the indices most strongly associated with hypertension. Our findings support these studies in that they show that this metabolic burden is synchronised not only with circadian disruption but also directly with the severity of blood pressure variability [19]. Furthermore, some studies have shown that the TyG-BMI index has high predictive value for nocturnal hypertension, which is consistent with the low rate of systolic blood pressure reduction (%7.71) observed in patients with Stage 2 hypertension in our study [20].

The echocardiographic data obtained from our study have clearly demonstrated that the left atrial diameter (LAd) is an independent structural marker for predicting the reverse-dipper pattern. Similar studies in the literature report that the reverse-dipper pattern is closely associated with impaired left atrial phasic function, independent of other clinical parameters [7]. In line with this, the wider LAd measurements observed in the Non-Dipper (36.48 mm) and Reverse-Dipper (35.57 mm) groups in our study strongly support the pathological link between circadian rhythm disruption and the early-stage structural cardiac remodelling process.

On the other hand, Kimura et al. (1999) reported that morphological changes, such as RWT (relative wall thickness) and IVSD (interventricular septal diameter), begin even at ‘High-Normal’ blood pressure levels where clinical hypertension has not yet developed [21]. In our findings, too, the fact that the RWT (0.41) and IVSD (10.71 mm) values in the ‘High’ blood pressure group were found to be significantly higher than those in the Normal group serves to complement and corroborate this ‘early remodelling’ theory in the literature.

The circadian distribution in our study (*n* = 278 non-dippers, 51.6%) was consistent with the prevalence of non-dipping reported in the literature. In particular, the fact that the Reverse-Dipper group (50.32 years) was significantly older than the other groups may be interpreted as a reflection of impaired autonomic nervous system regulation associated with ageing. Some other studies have highlighted that non-dipper patients are older than dippers and that this is associated with increased arterial stiffness and impaired left atrial function [22]. The fact that age and LAd emerged as strong predictors of reverse-dipping in our regression model suggests that, in clinical practice, hypertensive patients who are of advanced age and have left atrial dilatation should be monitored more closely for circadian rhythm disturbances.

Furthermore, although our study showed a numerical upward trend in inflammatory indices such as NLR, SII and AISI in relation to the stage of hypertension and circadian patterns, this did not reach statistical significance (*p* > 0.05). This may indicate that inflammation plays a more pronounced role in the more advanced stages of hypertension or in its chronic course. A similar finding has been observed in some studies in the literature; it has been argued that metabolic parameters are more sensitive than inflammatory parameters in predicting circadian rhythm disturbances [10]. In summary, the stage of rising blood pressure and disrupted circadian rhythm is characterised by an increased TyG index, AIP and left atrial dilatation. In particular, the fact that the reverse-dipping pattern is more common in elderly patients with left atrial dilatation highlights the need for more aggressive management of cardiovascular risk in this patient group.

## 5. Study Limitations

Our study has some limitations. First, due to its retrospective nature, the left ventricular mass index could not be evaluated. However, we successfully addressed geometric cardiac remodeling by reporting the relative wall thickness, which averaged 0.40 ± 0.06 in our cohort, reflecting a well-characterized hypertensive remodeling pattern. Second, the single-center design and the inclusion of only the Turkish population may limit the generalizability of our findings. Finally, metabolic risk and insulin resistance were assessed using surrogate markers such as the TyG index and AIP, rather than the gold-standard hyperinsulinemic–euglycemic clamp technique.

## 6. Conclusions

This study comprehensively demonstrates the metabolic, inflammatory, and structural cardiac relationships between blood pressure stages and circadian blood pressure profiles in patients with a preliminary diagnosis of hypertension. Our findings indicate that the metabolic risk burden increases significantly as blood pressure stages progress. Furthermore, circadian blood pressure profiles were found to exhibit a strong correlation with age and cardiac remodeling. In conclusion; the evaluation of practical metabolic markers, such as the triglyceride–glucose (TyG) index and AIP, alongside the presence of echocardiographic parameters—namely LVH indicating target organ damage and RWT indicating cardiac remodeling—possesses high prognostic and predictive value in the clinical management of hypertensive patients.

## Figures and Tables

**Table 1:** Demographic and baseline clinical characteristics of the study population.

Characteristics	Total Population (*n* = 539)
Age (years)	
Mean ± SD	46.49 ± 13.98
Median (Min–Max)	46.00 (17.00–82.00)
Gender, *n* (%)	
Female	330 (61.2%)
Male	209 (38.8%)
Clinical History, *n* (%)	
Diabetes Mellitus	74 (13.7%)
Coronary Artery Disease	70 (13.0%)

Note: Data are presented as Mean ± Standard Deviation or Frequency. Abbreviations: SD: Standard Deviation; Min: Minimum; Max: Maximum.

**Table 2:** Baseline biochemical and hematological characteristics of the study cohort.

Parameters	Total Population (*n* = 539)
Lipid Profile	
Total Cholesterol (mg/dL)	193.90 ± 38.26
Triglyceride (mg/dL)	162.76 ± 89.05
HDL-C (mg/dL)	46.24 ± 12.35
LDL-C (mg/dL)	115.60 ± 31.57
Metabolic & Renal	
Glucose (mg/dL)	103.56 ± 37.62
HbA1c (%)	5.85 ± 2.23
Urea (mg/dL)	31.13 ± 10.75
Creatinine (mg/dL)	0.77 ± 0.26
Albumin (g/L)	42.61 ± 4.50
Inflammatory & Hematological	
CRP (mg/L)	4.10 ± 4.60
WBC (×10^3^/µL)	8.05 ± 2.19
Hemoglobin (g/dL)	14.03 ± 1.80
Hematocrit (%)	42.73 ± 5.05
Platelets (×10^3^/µL)	282.06 ± 73.64
TSH (mIU/L)	1.85 ± 2.05

Note: Data are presented as Mean ± Standard Deviation. Abbreviations: HDL-C: High-density lipoprotein cholesterol; LDL-C: Low-density lipoprotein cholesterol; HbA1c: Glycated hemoglobin; CRP: C-reactive protein; WBC: White blood cell; TSH: Thyroid-stimulating hormone.

**Table 3:** Demographic and Clinical Characteristics of Participants According to AHA/ACC Stages.

Variable	Normal (*n* = 191)	High (*n* = 126)	Stage 1 HT (*n* = 164)	Stage 2 HT (*n* = 58)	Total (*n* = 539)	*p*-Value
Age, Mean ± SD	43.93 ± 14.55	49.06 ± 14.93	47.29 ± 11.94	47.10 ± 14.31	46.49 ± 13.98	0.010
Gender, *n* (%)						<0.001 ‡
Female	144 (%75.4)	66 (%52.4)	89 (%54.3)	31 (%53.4)	330 (%61.2)	
Male	47 (%24.6)	60 (%47.6)	75 (%45.7)	27 (%46.6)	209 (%38.8)	
Diabetes Mellitus, *n* (%)	26 (%13.6)	18 (%14.3)	19 (%11.6)	11 (%19.0)	74 (%13.7)	0.540 ‡
Previous CV History, *n* (%)	27 (%14.1)	16 (%12.7)	17 (%10.4)	10 (%17.2)	70 (%13.0)	0.569 ‡
Prevalence of LVH, *n* (%)	14 (%7.3)	29 (%23.0)	32 (%19.5)	20 (%34.5)	95 (%17.6)	<0.001 ‡

Abbreviations: HT: Hypertension, CV: Cardiovascular, LVH: Left Ventricular Hypertrophy. ‡ Pearson Chi-Square.

**Table 4:** Analysis of Age Distribution According to Circadian Blood Pressure Patterns.

Pattern Group	*N*	Age (Mean ± SD)	*p*-Value	Clinical Note
Dipper	135	42.15 ± 13.15	<0.001	Reference Group
Non-Dipper	404	47.94 ± 13.97		A wise old man from the Dipper
Reverse Dipper	126	50.32 ± 14.55	<0.001	Highest average age
Extreme Dipper	38	38.42 ± 13.01	<0.001	Lowest average age

Note: The statistical analyses were validated using the independent samples *t*-test and ANOVA.

**Table 5:** Summary of Echocardiographic Findings by AHA/ACC Stages and Circadian Pattern Groups.

Groups	IVSD (mm)	LVPWd (mm)	LAd (mm)	RWT
**AHA/ACC Stages**				
Normal (*n* = 191)	9.78	8.61	34.27	0.38
High (*n* = 126)	10.71	9.41	36.06	0.41
Stage 1 HT (*n* = 164)	10.59	9.45	35.29	0.41
Stage 2 HT (*n* = 58)	10.17	9.17	33.50	0.39
**Circadian Patterns**				
Dipper	10.11	8.76	33.21	0.38
Non-Dipper	10.85	9.70	36.48	0.41
Reverse-Dipper	10.49	9.27	35.57	0.40
Extreme-Dipper	10.10	9.00	34.24	0.39

Abbreviations: IVSD: Interventricular septal diastolic thickness; LVPWd: Left ventricular posterior wall diastolic thickness; LAd: Left atrial diameter; RWT: Relative wall thickness; HT: Hypertension.

**Table 6:** Comparative Analysis of Parameters According to AHA/ACC Stages.

Variable	Normal (*n* = 191)	High (*n* = 126)	Stage 1 HT (*n* = 164)	Stage 2 HT (*n* = 58)	*p* Value
TyG Index	8.79 ± 0.70	8.87 ± 0.61	8.99 ± 0.63	9.22 ± 0.78	0.002
AIP	0.38 ± 0.33	0.44 ± 0.35	0.48 ± 0.35	0.52 ± 0.33	0.004
Decrease in Systolic BP (%)	10.37 ± 6.90	8.72 ± 7.91	8.35 ± 7.73	7.71 ± 7.15	0.042
NLR	2.13 ± 1.17	2.16 ± 0.91	2.21 ± 1.18	2.43 ± 1.51	0.090
SII	525.8 ± 265.3	520.3 ± 204.4	563.3 ± 311.9	609.8 ± 395.0	0.076
AISI	352.7 ± 255.2	344.8 ± 174.4	385.8 ± 274.3	419.0 ± 344.0	0.291

Abbreviations: AIP, atherogenic index of plasma; AISI, aggregate index of systemic inflammation; BP, blood pressure; HT, hypertension; NLR, neutrophil-to-lymphocyte ratio; *p*, statistical significance value; SII, systemic immune-inflammation index; TyG Index, triglyceride–glucose index. A *p*-value of < 0.05 was considered statistically significant.

**Table 7:** Multivariate logistic regression analysis of factors influencing the prediction of different circadian patterns.

Independent Variables	Extreme-Dipper		Non-Dipper		Reverse-Dipper	
	OR (%95 CI)	*p*	OR (%95 CI)	*p*	Score/OR	*p*
LAd (mm)	1.19 (1.06–1.34)	0.003	1.11 (1.04–1.18)	0.001	13.69	<0.001
AIP	0.04 (0.00–0.54)	0.016	0.85 (0.23–3.12)	0.829	3.72	0.054
Decrease in Systolic BP (%)	0.98 (0.96–0.99)	0.001	-	-	89.21	<0.001
TyG Index	3.45 (0.90–13.2)	0.070	1.17 (0.60–2.32)	0.638	2.94	0.086
AISI	1.00 (0.99–1.00)	0.409	1.00 (0.99–1.01)	0.666	5.12	0.024
NLR	1.25 (0.66–2.39)	0.482	0.75 (0.50–1.13)	0.139	0.06	0.797
SII	0.99 (0.99–1.00)	0.152	1.00 (0.99–1.00)	0.498	1.28	0.256
LVH	0.89 (0.30–2.62)	0.836	1.66 (0.83–3.32)	0.129	11.67	0.001

Abbreviations: AIP, atherogenic index of plasma; AISI, aggregate index of systemic inflammation; BP, blood pressure; CI, confidence interval; LAd, left atrial diameter; LVH, left ventricular hypertrophy; NLR, neutrophil-to-lymphocyte ratio; OR, odds ratio; *p*, statistical significance value; SII, systemic immune-inflammation index; TyG Index, triglyceride–glucose index.

## Data Availability

No new data were created or analyzed in this study. All information was obtained from publicly available literature.

## Abbreviations

The following abbreviations are used in this manuscript:

ABPM	Ambulatory Blood Pressure Monitoring
AIP	Atherogenic İndex of Plasma
AISI	Aggregate Index of Systemic Inflammation
BP	Blood Pressure
CRP	C-Reactive Protein
DM	Diabetes Mellitus.
DBP	Diastolic Blood Pressure
HT	Hypertension
HDL-C	High-Density Lipoprotein Cholesterol
HbA1c	Glycated Hemoglobin
IVSD	İnterventricular Septal Thickness in Diastole,
LAd	Left Atrial Diameter
LDL-C	Low-Density Lipoprotein Cholesterol
LVEDD	Left Ventricular End-Diastolic Diameter
LVEF	Left Ventricular Ejection Fraction
LVH	Left Ventricular Hypertrophy
LVPWd	Left Ventricular Posterior Wall Diameter in Diastole.
NLR	Neutrophil-to-Lymphocyte Ratio
RWT	Relative Wall Thickness
SBP	Systolic Blood Pressure
SII	Systemic Immune-Inflammation Index
TyG İndex	Triglyceride–Glucose İndex
